# Medical Conditions, Oral Health Practices, and Barriers to Treatment among Patients Visiting a Teaching Dental Hospital in Eastern Saudi Arabia

**DOI:** 10.1155/2022/4495757

**Published:** 2022-02-04

**Authors:** Faisal A Alonaizan, Khalid Almas, Muhammad Ashraf Nazir, Dalal Almazrou, Manar Alzamil, Mohammed A. AlOlyani

**Affiliations:** ^1^Department of Restorative Dental Sciences, College of Dentistry, Imam Abdulrahman Bin Faisal University, Dammam 31441, P. O. Box 1982, Saudi Arabia; ^2^Department of Preventive Dental Sciences, College of Dentistry, Imam Abdulrahman Bin Faisal University, Dammam 31441, P. O. Box 1982, Saudi Arabia; ^3^Clinical Affairs Department, College of Dentistry and Dental Hospital Imam Abdulrahman Bin Faisal University, Dammam 31441, P. O. Box 1982, Saudi Arabia

## Abstract

**Objective:**

To assess the prevalence of medical conditions, oral hygiene practices, and dental visits among patients who attended a teaching dental hospital in Dammam, Saudi Arabia. *Materials & Methods*. This retrospective cross-sectional study used patient records from 2009 to 2015 from the dental hospital of the College of Dentistry Imam Abdulrahman Bin Faisal University, Dammam. Patients' demographics, medical history, oral hygiene practices, reasons for attending the facility, attendance patterns, and smoking habits were studied.

**Results:**

The study included 1502 records of patients with 65.1% of males and 34.9% of females. The prevalence of medical conditions was 25.7% in the study. The most common medical conditions included diabetes mellitus (7.2%), hypertension (6.5%), and anemia (4.7%). Only 21.8% reported visiting the dental hospital in the past one year. The prevalence of smoking was 16.7%, and this did not differ significantly between healthy and medically compromised patients (*P*=0.165). Fillings were the most common (21.6%) reason for visiting a dental hospital, followed by treatment for periodontal problems (12.9%) and oral lesions (12.6%), whereas treatment for braces (orthodontics) was the least common (5%) reason for visiting the hospital. The reasons for visiting the hospital did not differ significantly between healthy and medically compromised patients (*P* > 0.05). The three most common barriers to dental visits included long waiting time (18.1%), fear of dental treatment (14.4%), and difficulty in getting an appointment (11.3%).

**Conclusion:**

The study showed that dental patients had a high prevalence of medical conditions. Diabetes mellitus was the most prevalent problem. Most patients visited the dental hospital to receive restorative treatment, and a long waiting time was the most common barrier to dental visits. Public health measures should be taken to improve the general health and oral care of patients.

## 1. Introduction 

Dental professionals are confronted with the challenge of treating an increasingly large number of medically compromised patients in their dental practices [1]. Chronic systemic conditions in these patients may hinder the provision of quality dental care and may lead to adverse consequences including medical emergencies [2]. For instance, a patient with a prosthetic heart valve may develop bacterial endocarditis if antibiotic prophylaxis is not prescribed and adequate oral hygiene is not maintained [3]. Proper recording of medical history is important for the effective management of patients because medical conditions in many patients who are present for dental treatments may be ignored by dental professionals because they may look healthy [4]. Moreover, a strong relationship between systemic conditions and oral diseases that is consistently reported in the literature underscores the need for increased awareness about medically compromised patients [5–8].

Evidence from previous studies shows a high prevalence of medical problems among patients visiting dental care facilities in different parts of the world [4, 9–11]. Al-Bayaty et al. reported medical conditions in 42% of the patients who attended the emergency clinic of a dental school in the West Indies [9]. Similar prevalence estimates (38%) were reported by Aggarwal et al. in a study of dental patients in a teaching dental institute in India [4]. A recent study by Frydrych et al. identified medical problems in 86% of dental patients in Australia, and the most common medical problems included cardiovascular disease, allergy, and mental disorders [11]. On the other hand, Oyetola et al. reported the occurrence of medical conditions in 11.7% of patients in Nigeria [10]. In Saudi Arabia, two studies evaluated the prevalence of medical conditions among patients attending periodontal clinics in teaching dental hospitals in the Riyadh and Asir regions [12, 13]. Almas and Awartani indicated that 10% of patients had medical conditions in the Riyadh region [12], whereas Javali et al. reported medical conditions in 40.2% of patients in the Asir region [13].

Certain dental procedures are contraindicated in some medically compromised patients, and some medical conditions require the use of medication or special arrangements prior to the provision of dental care [7, 14]. Therefore, dental students, dental practitioners, and dental staff are required to have adequate knowledge about chronic systemic conditions, proper management of medically compromised patients, and their referral to physicians [8, 15]. It is also important that dental professionals should be aware of the oral hygiene behaviors and tobacco consumption practices of their patients so they can play an important role in the prevention of oral diseases and promotion of optimal oral health. They should also raise awareness about the positive oral health outcomes associated with routine dental visits among their patients. However, there is a lack of data concerning the medically compromised patients in the Eastern Province of Saudi Arabia. In addition, there was a need to investigate the frequency of dental attendance and dental treatment reasons for visiting the dental hospital [12, 13, 16]. Therefore, the study aimed to assess the prevalence of medical conditions, oral hygiene practices, and dental visits among patients who attended a teaching dental hospital in Dammam, Saudi Arabia.

## 2. Materials and Methods

### 2.1. Study Design and Subjects

This retrospective cross-sectional study included records of male and female patients attending the dental hospital of the College of Dentistry at the Imam Abdulrahman Bin Faisal University (IAU), Dammam, Saudi Arabia. Patient records from the years 2009 to 2015 were reviewed for demographic information, medical history, oral hygiene practices, dental visit behaviors, and smoking habits. A sample size of 1611 records was calculated using a 95% confidence interval, 2% margin of error, 30% response distribution, and approximate population size (≈8,000). A convenience sample of patient records was used for the present study. Patient records were excluded from the study if there was inaccurate or incomplete information about study variables. In addition, the records of children and adolescents (≤ age of 18 years) were excluded from the study.

### 2.2. Measurement of Study Variables

Two researchers (DA and MA) involved in the recording of data went through training and calibration. These calibrated examiners then reviewed selected patient charts and recorded data in a structured chart that included demographic information (age and gender), reasons for visiting the dental hospital, medical conditions, patterns of dental visits, smoking, and oral hygiene practices. Gum problems (periodontal disease), fillings, braces, oral surgery, crowns and bridges, root canal treatment, dentures, and oral lesions were recorded as the reasons for attending a dental hospital. Medical history included information about cardiac disease, hypertension, diabetes, anemia, renal disease, liver disease, asthma, gastric disease, allergy to medication, and sickle cell disease. The frequency of visiting the dental hospital and barriers to dental attendance were recorded under patterns of dental visits. Oral hygiene practices included the use of toothbrush and floss and smoking habits.

### 2.3. Ethical Considerations

The hospital administrator authorized the collection, analysis, and publication of data from patients' records. Patient charts and patient names were coded to protect confidentiality and privacy. The study was conducted in accordance with the guidelines of the Declaration of Helsinki. The Strengthening the Reporting of Observational Studies in Epidemiology (STROBE) statement was used for the reporting of the study [17].

### 2.4. Statistical Analysis

Data from 1502 patients' records were entered into Microsoft Excel (2010) and analyzed using the Statistical Package for Social Sciences (IBM SPSS Statistics for Windows, version 22.0. Armonk, NY: IBM Corp). Descriptive data were presented in the form of frequencies, proportions, means, and standard deviations. The chi-square test was performed to compare medical conditions between male and female participants. Similarly, oral hygiene behaviors and reasons for dental visits were compared between healthy and medically compromised participants using chi-square tests. Statistical significance was set at *P* value <0.05.

## 3. Results

The study included charts of 978 male (65.1%) and 524 female (34.9%) patients. Half the sample (48.6%) was below 30 years of age, and medical conditions were recorded in 25.7% of patients (Table 1).

**Figure 1:**
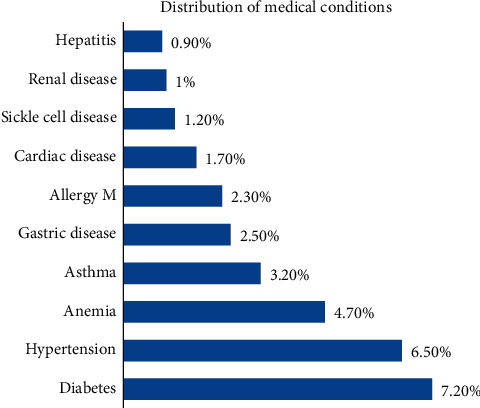
Distribution of medical conditions among patients.

Diabetes (*N* = 108, 7.2%), hypertension (*N* = 98, 6.5%), and anemia (*N* = 70, 4.7%) were the most commonly reported conditions. The least commonly reported conditions included hepatitis (*N* = 13, 0.90%), renal disease (*N* = 15, 1%), and sickle cell disease (*N* = 18, 1.20%) (Figure 1).

The three most commonly distributed medical conditions were compared between male and female patients and are presented in Table 2. It was found that a significantly greater proportion of males than females had diabetes (*P*=0.047) and hypertension (*P*=0.031).

Toothbrushing was observed in 67.5% and dental flossing in 12.4% of the sample. No statistically significant differences were observed between healthy and medically compromised patients with regards to toothbrushing (*P*=0.091) and flossing (*P*=0.714). There were 16.7% of smokers in the study, and smoking did not differ significantly between healthy and medically compromised patients (*P*=0.165) (Table 3).

The patients reported visiting the dental hospital within 0–6 months (11.1%), 7–12 months (10.7%), and after 12 months (49.5%). About one quarter (28.8%) of the sample had never visited the hospital before. No statistically significant differences were observed between healthy and medically compromised patients with regard to the frequency of visiting the dental hospital (*P*=0.758) (Table 4).

Fillings (tooth restorations) were the most common reason (21.6%) for visiting the dental hospital, followed by periodontal problems (12.9%) and oral lesions (12.6%). Braces (orthodontics) were the least common reason for visiting the hospital (5%). The reasons for visiting the hospital did not differ significantly between healthy and medically compromised patients (*P* > 0.05) (Table 5).

**Figure 2:**
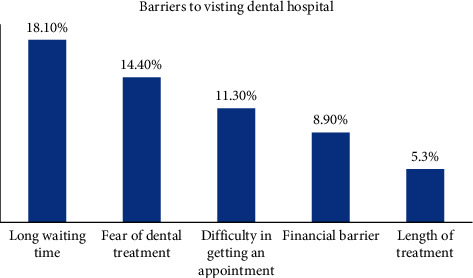
Barriers to visiting the dental hospital among patients.

The three most common barriers to dental visits to the hospital included long waiting time (18.1%), fear of dental treatment (14.4%), and difficulty in getting an appointment (11.3%). Length of the treatment was the least commonly reported (5.3%) barrier to visiting the dental hospital (Figure 2).

## 4. Discussion

The present study evaluated the distribution of medical problems among patients visiting a teaching dental hospital in the eastern part of Saudi Arabia. The study showed that 25.7% of the patients had medical conditions. In contrast, a recent study by Javali et al. [13] reported a higher prevalence of medical conditions (40.2%) in patients who visited a teaching dental hospital for periodontal treatment in the Asir region (southwest) of the country [13]. On the other hand, Almas and Awartani (2003) reported the distribution of systemic diseases in 10% of the patient population after analyzing patient records from periodontal clinics at the College of Dentistry, King Saud University, Riyadh, Saudi Arabia [12]. The exponential increase in diabetes mellitus and its complications over the last decades may account for a higher prevalence of medical conditions in the present study compared with the estimates by Almas and Awartani in 2003 [12, 18, 19]. International literature indicates variations in the prevalence estimates of medical conditions in dental patients in different countries [4, 7–9, 16–18]. The prevalence of medical conditions in dental patients was 11.7% in Nigeria [10], 12.2% in Thailand [20], 27.7% in Ireland [21], 38% in India [4], 42% in West Indies [9], 52.5% in the U.S. [22], and 86% in Australia [11].

Diabetes and hypertension were the most common conditions in the present study. These findings are in accordance with the results of previous similar studies from Saudi Arabia [12, 13]. The analysis of data from the World Health Organization and the International Diabetes Federation revealed that Saudi Arabia had the highest prevalence of diabetes (14.4% and 20%, respectively) among high-income countries [23, 24]. The literature also reports the existence of diabetes in 7 million and prediabetes in 3 million people in Saudi Arabia [19]. Hypertension and dyslipidemia among diabetic patients are the most common risk factors for cardiovascular diseases, and 70–75% of patients with coronary artery disease also have diabetes or abnormal glucose levels [25]. The studies from India and the West Indies also showed that diabetes and hypertension were the most prevalent conditions among dental patients [4, 9]. On the other hand, gastrointestinal disease and bleeding tendencies were common conditions among dental patients in Jordan [16]. The most common medical conditions in patients attending dental clinics in Western Australia included cardiovascular disease, allergies, and mental disorders [11]. Similarly, drug allergies and cardiovascular disease were the most frequently reported in patients in the U.S. [22]. The pattern of medical conditions identified in the present study can help dental professionals better manage patients with medical problems to avoid medical emergencies and other adverse outcomes in dental practice in the Eastern Province of Saudi Arabia. It is known that the patients with medical conditions are more likely to experience medical emergencies [26]. It was also reported that 35% of the patients who suffered medical emergencies in dental settings had underlying systemic diseases [27].

Oral lesions are common, and they range from ulcerations, infections, hyperpigmentation, and benign to malignant neoplasms [28]. This is depicted in the present study where 12.6% of participants reported visiting the dental hospital because of oral lesions. The literature indicates that systemic diseases are associated with oral lesions [29, 30]. For instance, oral ulceration may be a sign of underlying systemic conditions [30]. Likewise, certain chronic conditions such as dyslipidemia and asthma are independently associated with oral leukoplakia [29]. Given the challenges of diagnosing and treating oral lesions due to remarkable similarities in their clinical appearance, it is crucial for clinicians to have a clear understanding of the distribution and clinical presentations of oral lesions for the provision of quality oral care.

The present study showed that most patients visited the dental hospital for fillings (tooth restorations) and treatment for periodontal problems. The high occurrence of caries and periodontal disease in Saudi Arabia may account for a greater need for restorative treatment for untreated caries and periodontal therapy in our sample of the patients. In Saudi Arabia, a high prevalence of dental caries has been observed in adult populations with estimates ranging from 68.5% to 98% [31]. Similarly, poor periodontal health is also a significant oral health problem among adults in the country. A recent review reported a lack of periodontal health in 50% of the adult population in Saudi Arabia and showed a strong association with diabetes and tobacco use [32]. In addition, the existence of a strong bidirectional relationship between periodontal disease and diabetes can further worsen oral and systemic health [33].

Dental services utilization behaviors are known to affect oral health, and routine dental visits are associated with lower caries experience, tooth loss, dental pain, and improved oral health-related quality of life [34, 35]. A previous study reported that only 9.6% of participants attended dental office within six months in the Eastern Province of Saudi Arabia [36]. Similar trends of low dental attendance were observed in the present study where only 11.1% visited the hospital within 6 months and 28.8% had never visited the dental hospital before. The study did not find significant differences in dental visiting patterns between medically compromised and healthy patients. Given the importance of routine dental visits for positive oral health outcomes and the high prevalence of medical conditions in dental patients, the patterns of dental attendance should be investigated in medically compromised patients in large multicenter studies in the future.

Oral health depends upon the interaction of factors related to individuals' perceptions, attitudes, and behavior [37]. Appropriate oral hygiene is required for the prevention of oral diseases and the maintenance of optimal oral health. Poor oral hygiene practices were observed in our sample where one-third of patients did not use toothbrush and only a small proportion of participants used dental floss. Moreover, a considerable proportion of participants were smokers in the present study. Similar trends of oral hygiene practices and smoking habits were also observed in a study of adolescents from Dammam, Saudi Arabia, where toothbrushing was reported in 64.5% and smoking in 20.2% of the participants [38]. The literature also indicates the lack of importance of oral health in Saudi populations despite the high prevalence of oral diseases in the country [37].

There were certain limitations to the present study. First, self-reported information about study variables may lead to bias in the present research. Second, data were collected from records of patients visiting a teaching dental hospital in the Eastern Province; hence, the generalizability of study results to dental patients in other regions of Saudi Arabia should be avoided. Third, this study could not evaluate routine dental visits because dental records lacked this information. Fourth, the type and frequency of oral lesions were not investigated in the present study, despite the fact that medical conditions are associated with oral lesions.

## 5. Conclusion

This study showed a high prevalence of medical conditions in dental patients. Diabetes mellitus was the most prevalent problem. Most patients visited the dental hospital to receive restorative treatment, and a long waiting time was the most common barrier to dental visits. The study findings call for integration between dental and medical professionals for improved general health of patients. Dental professionals should take measures to reduce barriers to oral care for positive oral health outcomes.

## Figures and Tables

**Table 1:** Descriptive data analysis of patients (*N* = 1502).

Variables	Frequency	Percentages
Gender		
Male	978	65.1
Female	524	34.9

Age (*N* = 1434)		
Less than 30 years	697	48.6
30–40 years	311	21.7
41–50 years	197	13.7
More than 50 years	229	16.0

Health status		
Healthy	1116	74.3
Medically compromised	386	25.7

**Table 2:** Comparison of medical conditions between male and female patients (*N* = 1502).

Medical condition	Male *N* (%)	Female *N* (%)	*P* value
Diabetes			
Yes	80 (74.1)	28 (25.9)	0.047^*∗*^
No	893 (64.1)	501 (35.9)	

Hypertension			
Yes	54 (55.1)	44 (44.9)	0.031^*∗*^
No	924 (65.8)	480 (34.2)	

Anemia			
Yes	38 (54.3)	32 (45.7)	0.052
No	940 (65.6)	492 (34.4)	

^*∗*^Statistically significant.

**Table 3:** Comparison of oral hygiene practices and smoking between healthy and medically compromised patients (*N* = 1502).

Frequency of dental visits	Total *N* (%)	Healthy *N* (%)	Medically compromised *N* (%)	*P* value
Toothbrushing				
Yes	1014 (67.5)	740 (73)	274 (27)	0.091
No	488 (32.5)	376 (77)	112 (23)	

Dental flossing				
Yes	186 (12.4)	138 (75.4)	45 (24.6)	0.714
No	1316 (87.6)	975 (74.1)	341 (25.9)	

Smoking				
Yes	251 (16.7)	193 (77.8)	55 (22.2)	0.165
No	1251 (83.3)	921 (73.6)	330 (26.4)	

**Table 4:** Comparison of frequency of visiting the hospital between healthy and medically compromised patients (*N* = 1067).

Frequency of dental visits	Total *N* (%)	Healthy *N* (%)	Medically compromised *N* (%)	*P* value
0–6 months	118 (11.1)	84 (71.2)	34 (28.8)	0.758
7–12 months	114 (10.7)	87 (76.3)	27 (23.7)	
More than 12 months	528 (49.5)	393 (74.4)	135 (25.6)	
Never visited before	307 (28.8)	233 (75.9)	74 (24.1)	

**Table 5:** Comparison of reasons for visiting the dental hospital between healthy and medically compromised patients (*N* = 1502).

Reasons for visiting a dental hospital	Total *N* (%)	Healthy *N* (%)	Medically compromised *N* (%)	*P* value
Periodontal problems				
Yes	194 (12.9)	149 (76.8)	45 (23.2)	0.393
No	1308 (87.1)	967 (73.9)	341 (26.1)	

Fillings (tooth restorations)				
Yes	325 (21.6)	240 (73.8)	85 (26.2)	0.832
No	1177 (78.4)	876 (74.4)	301 (25.6)	

Braces (orthodontics)				
Yes	75 (5)	53 (70.7)	22 (29.3)	0.460
No	1427 (95)	1063 (74.5)	364 (25.5)	

Oral surgery				
Yes	130 (8.7)	92 (70.8)	38 (29.2)	0.335
No	1372 (91.3)	1024 (74.6)	348 (25.4)	

Crowns and bridges				
Yes	102 (6.8)	69 (67.6)	33 (32.4)	0.111
No	1400 (93.2)	1047 (74.8)	353 (25.2)	

Root canal treatment				
Yes	133 (8.9)	101 (75.9)	32 (24.1)	0.651
No	1369 (91.1)	1015 (74.1)	354 (25.9)	

Dentures				
Yes	94 (6.3)	73 (77.7)	21 (22.3)	0.441
No	1408 (93.7)	1043 (74.1)	365 (25.9)	

Oral lesions				
Yes	189 (12.6)	133 (70.4)	56 (29.6)	0.186
No	1313 (87.4)	983 (74.9)	330 (25.1)	

## Data Availability

The SPSS data file of this study is available from the corresponding author upon request.
